# Suicidality in individuals with Prader-Willi syndrome: a review of registry survey data

**DOI:** 10.1186/s12888-021-03436-3

**Published:** 2021-09-06

**Authors:** Analise Peleggi, Jessica Bohonowych, Theresa V. Strong, Lauren Schwartz, Soo-Jeong Kim

**Affiliations:** 1 Department of Psychiatry and Behavioral Sciences, University of Washington, Seattle, Washington USA; 2 Foundation for Prader-Willi Research, Walnut, California USA; 3 Department of Rehabilitation Medicine, University of Washington School of Medicine, Seattle, Washington USA; 4 Seattle Children’s Autism Center, Seattle, Washington USA

**Keywords:** Prader-Willi syndrome, PWS, Suicidality

## Abstract

**Introduction:**

Prader–Willi syndrome (PWS) is a rare, genetic, neurodevelopmental syndrome associated with hyperphagia and early onset obesity, growth and sex hormone insufficiencies, mild-to-moderate intellectual disability, and behavioral challenges such as compulsivity, anxiety, skin picking, social skills deficits and temper outbursts. Given high rates of psychiatric comorbidity and potential risk factors for suicide in PWS, this study sought a first estimate of the prevalence of suicidal ideation (SI) and attempts (SA) in the PWS population and any characteristics associated with suicidality in this population.

**Methods:**

Using the Global Prader-Willi Syndrome Registry, we included all participants who had answered a question about SI. We examined the most recent data from the surveys about social, economic, and demographic factors, genetic subtype, and psychiatric symptoms and treatments. A chi-square analysis was used to compare registry participants who reported SI to those without reported SI.

**Results:**

From 750 included survey respondents, 94 (12.5%) endorsed some history of SI. Of these, 25 (26.6%) also reported a history of SA, with an average age of 16.25 years at their first attempt. Those with a history of SI were predominantly male and adult age, and had higher rates of aggression and psychiatric comorbidities, therapies, and medications.

**Conclusions:**

This study indicates that the rate of SI and SA in PWS is comparable to the general population, and that suicide attempts in PWS typically begin in middle-teenage years. Despite unique challenges, individuals with PWS and their caregivers should be included in screens and psychoeducation for suicide and mental health concerns.

## Introduction

Suicide accounts for 1.4% of premature deaths worldwide and affects all ages (Bachmann, 2018). More than 47,500 individuals died by suicide in 2019 alone [1]. Suicidal ideation (SI) and suicidal attempts (SA) are important predictors for repeat attempts as well as subsequent death [2]. Identifying rates of SI and SA, as well as the factors that impact risk in certain groups can help target interventions to reduce harm and loss of life due to suicide. Prevalence estimates of SI and SA vary significantly across population and period, with rates increasing in recent years. The national rate of suicide in the US has increased by 30% from 2000 to 2016 [3]. When examining race, Ivey-Stephenson et al. found that white non-Hispanic students had higher prevalence estimates of SI (19.1%) compared to other groups, while black non-Hispanic students had higher estimates of SA (11.8%) [4]. For all ages, the majority of suicides worldwide appear related to psychiatric disorders. Among those, depression, substance use, and psychosis constitute the most relevant risk factors, while anxiety, personality-, eating- and trauma-related disorders also significantly add to unnatural causes of death compared to the general population [5]. These studies offer valuable insights for the general population. For some unique cohorts, however, much about suicidality is still unknown.

Prader–Willi syndrome (PWS; OMIM #176270) is a rare genetic condition with a prevalence rate between 1/15,000 and 1/20,000 [6]. The genetic mechanism for PWS involves absence of expressed genes from the paternally inherited 15q11–13 region of chromosome 15 via deletion (only having one maternally inherited copy of 15q11-q13 region without paternally inherited copy of 15q11-q13 region), maternal uniparental disomy (UPD, having two copies of maternally inherited 15q11-q13 region in the absence of paternally inherited copy of 15q11-q13 region), or defects in the imprinting center [7, 8]. PWS is a complex neurobehavioral condition affecting multiple systems. Clinical characteristics of PWS include hypotonia with poor suck and poor weight gain in infancy, early childhood obesity, hyperphagia, hypogonadism, and growth hormone insufficiency causing short stature [6, 9]. In addition, persons with PWS often present with cognitive and neurobehavioral disturbances, such as mild to moderate intellectual disability and behavioral difficulties such as impulsivity, compulsivity, anxiety, difficulty with change in routine, and self-injurious behavior (e.g., skin picking, nail pulling, etc.) [10–13]. Individuals with PWS appear to struggle more with severe behavioral problems compared to others with similar levels of intellectual disability [12]. Some studies suggest a higher incidence of social communication deficits and/or psychosis in those with UPD versus deletion [12, 14–16].

Suicidality among persons with intellectual developmental disorders (IDD) are less well studied. Contrary to a belief that IDD could be protective against suicidality, recent studies, albeit few, have not supported this assumption [17]. For example, Walters et al., reported 21% of 90 youth admitted to a dual diagnosis specialty unit at a children’s psychiatric hospital had suicidal behavior [18]. Hardan and Sahl conducted a retrospective study of 233 youth with developmental and comorbid psychiatric disorders, and reported 20% of youth had SI and/or SA [19]. Lunsky also reported that 34% of 98 adults with IDD had SI from “sometimes” to “most of the time” [20]. While above reports were derived from relatively small and heterogenous samples, they suggest that persons with ID, especially those with comorbid psychiatric disorders may be at even higher risk for developing suicidal behaviors [21].

Given the high rates of psychiatric comorbidity and intellectual disability as well as the emotional, social, and behavioral challenges, individuals with PWS may be at increased risk of suicide. However, no study has specifically examined the rate of suicidal thoughts, behaviors, or attempts among persons with PWS nor the factors that may be associated with suicidality in this population. Suicide can be potentially prevented with appropriate screening, identification of risk factors, and additional support for vulnerable individuals. Without insight into the elements associated with suicidality in individuals with PWS, however, effective intervention is challenging.

The primary aim of this study was to obtain prevalence estimates for suicidal thoughts and/or behaviors among individuals with PWS in the community, as reported in the Global Prader-Willi Syndrome Registry [22]. In addition, given higher rates of social communication deficits and psychosis among those with UPD genetic subtype, we hypothesized that suicidal thoughts and behaviors may be higher in those with UPD subtype as well as those with comorbid psychiatric disorders. We also sought to explore whether other demographic, environmental, and social or economic factors are associated with higher rates of suicidal behaviors.

## Methods

### Data source and approval

The authors AP and SK obtained approval for Access of De-Identified Data from the Global PWS Registry (https://pwsregistry.org/) [22, 23]. The present study reviewed a subset of *de-identified* data from the Global PWS Registry reported by individuals with PWS and their caregivers worldwide. The Global PWS Registry is a comprehensive and secure database developed in 2015 to accelerate research for PWS. Participants in the registry are invited to complete surveys about developmental history, medical needs and care, quality of life, social and demographic characteristics, and other aspects of living with PWS. Surveys are completed when participants join the registry, then participants are prompted to update information with additional surveys as needed. In this way, the Global PWS Registry is a living record that reflects the most recent data provided by participants at different points in time. The “subject” in the registry and in this study refers to the person with PWS. The “respondent” is the individual providing answers to registry surveys, which can be either the subject, themselves, or a caregiver.

Ethics approval: This study was reviewed and approved by Seattle Children’s Hospital Institutional Review Board (IRB). The Global PWS Registry was approved by the New England IRB. All methods were carried out in accordance with relevant guidelines and regulations.

### Study procedures and statistical analysis

Select variables for participants who provided an answer to the survey question: “Has subject ever expressed any thoughts/statements about suicide?” were collected from the Global PWS Registry. Subjects were included in the “Reported SI” group if they answered “Yes” to this question at any time and placed in the “No Reported SI” group if they answered either “No” or “Don’t know”. Data related to suicidality was first added to registry surveys in April 2015. Participants are invited to answer this question initially and could update answers as desired.

The IBM SPSS® Statistical Software, version 27, was used for data analyses. Descriptive statistics were used to report frequencies of different variables within Reported SI and No Reported SI groups. Chi-square statistics were used to compare answer proportions. Cramer’s V was also reported as a measure of association, i.e., effect size of each variables, which ranges 0 to 1. In addition, we also conducted binary logistic regression to examine independent effects of age (continuous) and sex (categorical) on the likelihood of having SI (binary) and SA (binary).

In this study, age was calculated by the registry using birth data and represents subject age at the time of this study’s data acquisition, December 17, 2020. While all data included in the PWS Global Registry and used in this research was the most up-to-date information provided by registry participants, it was collected via a number of surveys at different points in time. For subjects aged 18 and older at the time of our data collection, mean BMI was calculated (weight in kg/[height in cm]^2^) from most recently provided weight and height measurements. Using survey data about living status, we stratified subjects into groups reported to be living with or not living with respondent (mostly parents). Gross Annual Income, as reflected in the Global PWS Registry, was condensed into four income groups to assess general trends. Insurance Coverage was also distributed into relevant categories describing whether subjects were reported to have private, public (Medicare and/or Medicaid), other insurance, a combination of these options, or no insurance. If respondents did report SI in their survey, they were prompted to provide information about any suicide attempts, which were also examined in this study. Lifetime history of any self-injurious behavior (SIB) and aggressive/tantrum behaviors were compared between Reported SI and No Reported SI groups. SIB was noted to be present if respondents reported a history of common behaviors in PWS, e.g., skin-picking, nail and hair pulling, rectal digging, etc. on survey questions. We included registry data about whether subjects have had anxiety, depression, mania or bipolar disorder, and psychosis at any point in their lifetime, and additional information about whether these conditions were diagnosed or confirmed by a clinician. History of pharmacologic treatment was entered into the registry through two distributed surveys that both asked about certain medications in specific questions and invited respondents to list all past and current medications freely. The answers to these surveys were consolidated into variables reflecting history of treatment with “Any antidepressant”, “any antipsychotic”, “Any stimulant or Atomoxetine”, or “Any other psychiatric medication”. This last “Other” category included mood stabilizers, benzodiazepines, antiepileptics, and any other psychoactive medications not classically described by previous categories. We included all exposure to psychiatric medications or therapies that was recorded in the registry, regardless of reason for treatment.

## Results

A total of 750 respondents in the Global PWS Registry provided information about suicidal thoughts or statements. 55.7% of subjects were youth (age range 3 to 17 yrs.) with PWS, whereas 45.3% were 18 years and older. Females were 51.3% and males were 48.7% of the total study cohort. 94 (12.5%) respondents answered “Yes” to a history of SI. 25 subjects (26.6% of those with reported SI, or 3.3% of total sample) were reported to have attempted suicide. The mean age of onset for first SA was 16.25 years.

Chi-square analyses revealed that compared to those without reported SI, subjects with reported SI were significantly older, primarily age 22 and above, and predominantly male. The rates of SI in youth/young adults (6–21 years) vs. adults (≥22 years) were 7.7% vs. 22.8%, respectively, whereas the rates of SA in those between 13 and 21 years (no reported SA in children younger than 13 in this sample) vs. those who are 22 years and older were 2.9% vs. 6.8%, respectively (Table 1). Binary logistic regression analyses revealed that both age and sex were statistically significant predictors for reported SI [Age (Wald 29.281, df = 1, *p* < 0.001, Sex (Wald 5.086, df-1, *p* = 0.024)] and SA [Age (Wald 15.280, df = 1, *p* < 0.001, Sex (Wald 6.835, df-1, *p* = 0.009)].

**Table 1:** Demographic Characteristics of Subjects with Reported SI vs. Subjects with No Reported SI

		Reported SI	No Reported SI	Total Count	Pearson’s X^2^ (df), *p*-value	Cramer’s V
		Count (%)	Count (%)	Count (%)		
Age Group (years)	3 to 5	0 (0)	7 (1.1)	7 (0.9)	49.790 (4), < 0.001	
	6 to 12	6 (6.4)	**215 (32.8)**	221 (29.5)		
	13 to 17	15 (16)	**167 (25.5)**	182 (24.3)		0.258
	18 to21	16 (17)	74 (11.3)	90 (12)		
	22 and older	**57 (60.6)**	193 (29.4)	250 (33.3)		
Sex	Female	38 (40.4)	**347 (52.9)**	385 (51.3)	5.118 (1), 0.024	0.083
	Male	**56 (59.6)**	309 (47.1)	365 (48.7)		
Race	White	81 (86.2)	555 (84.6)	636 (84.8)	2.307 (5), NS	0.055
	Multi-race	5 (5.3)	46 (7)	51 (6.8)		
	Black	3 (3.2)	13 (2)	16 (2.1)		
	Asian	1 (1.1)	17 (2.6)	18 (2.4)		
	Native or Pacific Islander	1 (1.1)	3 (0.5)	4 (0.5)		
	Unknown	3 (3.2)	22 (3.4)	25 (3.3)		
Ethnicity	Non-Hispanic	68 (72.3)	454 (69.2)	522 (69.6)	0.435 (2), NS	0.024
	Hispanic	5 (5.3)	43 (6.6)	48 (6.4)		
	Unknown	21 (22.3)	159 (24.2)	180 (24)		
Genetic Subtype	Deletion	47 (50)	320 (48.8)	367 (48.9)	2.700 (2), NS	
	Uniparental disomy	25 (26.6)	220 (33.5)	245 (32.7)		0.066
	Other/Unknown	22 (23.4)	116 (17.7)	138 (18.4)		

No Reported SI includes either “No” or “Don’t Know” responses; it does not include subjects who did not respond

Bold cells are those significant by post hoc test. NS: *p*-value greater than 0.05. Age is age of survey subject as of data collection, 12/17/2020. Unknown includes both “Don’t know” and no provided answer. Cramer’s V: Measure of Association (Effect size ranging 0 to 1)

There were no significant differences in race, ethnicity, or genetic subtype between the two groups. BMI (calculated for subjects 18 and older only) was not different between groups, either (35.4 ± 11.7 in Reported SI vs. 34.4 with ±11.4 in No Reported SI). The majority of respondents for both groups were parents of the individual with PWS. Subjects without reported SI were more likely to be living with their survey respondent. A greater proportion of subjects with lifetime SI are neither in school nor employed; those with no reported SI were primarily in school. Lack of insurance coverage appeared to be associated with reported SI in our initial analysis, however frequencies were too small for reliable Chi-square results. (Table 2.)

**Table 2:** Life, Functioning, and Insurance Coverage in relation to Reported SI History

		Reported SI	No Reported SI	Total Count	Pearson’s X2 (df), *p*-value	Cramer’s V
		Count (%)	Count (%)	Count (%)		
Respondent Relationship to Subject	Parent (biologic, adoptive, step)	85 (90.4)	597 (91)	682 (90.9)	2.258 (5), NS	
	Legal Guardian	1 (1.1)	13 (2)	14 (1.9)		
	Grandparent	0	5 (0.8)	5 (0.7)		0.055
	Brother/Sister	1 (1.1)	3 (0.5)	4 (0.5)		
	Self	0	1 (0.2)	1 (0.1)		
	Unknown	7 (7.4)	37 (5.6)	44 (5.9)		
Respondent Living with Subject	Yes	67 (71.3)	**549 (83.7)**	616 (82.1)	9.633 (2), 0.008	0.113
	No	**18 (19.1)**	62 (9.5)	80 (10.7)		
	Unknown	9 (9.6)	45 (6.9)	54 (7.2)		
Country of residence	USA or Canada	90 (95.7)	601 (91.6)	691 (92.1)	1.934 (1), NS	0.051
	Other countries	4 (4.3)	55 (8.4)	59 (7.9)		
Subject Has a Best Friend	Yes	39 (41.5)	247 (37.7)	286 (38.1)	2.682 (2), NS	0.060
	No	25 (26.6)	230 (35.1)	255 (34)		
	Unknown	30 (31.9)	179 (27.3)	209 (27.9)		
Employment/ Education Status	Employed	12 (12.8)	56 (8.5)	68 (9.1)	40.646 (3), < 0.001	0.233
	In school	27 (28.7)	**376 (57.3)**	403 (53.7)		
	Not in school nor employed	**32 (34)**	80 (12.2)	112 (14.9)		
	Unknown	23 (24.5)	144 (22)	167 (22.3)		
Family Gross Income	Less than $45,000	15 (26.8)	127 (27.9)	142 (27.8)	7.079 (4), NS	0.097
	$45,000 – 84,999	18 (32.1)	116 (25.5)	134 (26.2)		
	$85,000 – 149,999	17 (30.4)	117 (25.7)	134 (26.2)		
	$150,000 and above	6 (10.7)	95 (20.9)	101 (19.8)		
	Unknown	38 (40.4)	201 (30.6)	239 (31.9)		
Insurance	Private Insurance Only	7 (7.4)	**152 (23.2)**	159 (21.2)	24.417 (6), < 0.001	0.180
	Private plus Public	31 (33)	164 (25)	195 (26)		
	Public Only	22 (23.4)	129 (19.7)	151 (20.1)		
	Public plus Other (not private)	2 (2.1)	15 (2.3)	17 (2.3)		
	Other Insurance only	3 (3.2)	41 (6.3)	44 (5.9)		
	Unknown	26 (27.7)	153 (23.3)	179 (23.9)		
	No Insurance	**3 (3.2)**	2 (0.3)	5 (0.7)		

No Reported SI includes either “No” or “Don’t Know” responses; it does not include subjects who did not respond. Bold cells are those significant by post hoc test. NS: *p*-value greater than 0.05. Unknown includes both “Don’t know” and no answer. Cramer’s V: Measure of Association (Effect size ranging 0 to 1)

Aggressive or tantrum behaviors were significantly more frequent in PWS subjects with reported SI compared to those without. Psychiatric comorbidities, both as endorsed conditions and confirmed diagnoses were all significantly more common in the Reported SI group, as well. A greater number of subjects with reported SI were stated to have a first-degree family member with a psychiatric diagnosis, although most of those who had no reported SI described psychiatric family history as “Unknown”. Notably, subjects with no endorsed history of SI also had a significantly lower prevalence of any SIB (Table 3.)

**Table 3:** Psychiatric Behavior, Diagnosis, and Family History in relation to Reported SI History

		Reported SI	No Reported SI	Total Count	Pearson’s X2 (df), p-value	Cramer’s V
		Count (%)	Count (%)	Count (%)		
Suicidal attempt	Reported	**25 (26.6)**	0 (0)	25 (3.3)	180.484 (1), < 0.001	0.491
	Not reported	69 (73.4)	**656 (100)**	725 (96.7)		
Self-injurious behavior	Yes	62 (66)	373 (56.9)	435 (58)	6.087 (2), 0.048	0.090
	No	6 (6.4)	**103 (15.7)**	109 (14.5)		
	Unknown	26 (27.7)	180 (27.4)	206 (27.5)		
Aggressive or Tantrum Behavior	Yes	**66 (70.2)**	372 (56.7)	438 (58.4)	7.226 (2), 0.027	
	No	2 (2.1)	44 (6.7)	46 (6.1)		0.098
	Unknown	26 (27.7)	240 (36.6)	266 (35.5)		
Anxiety, Reported	Yes	**82 (87.2)**	319 (48.6)	401 (53.5)	50.780 (2), < 0.001	
	No	7 (7.4)	**281 (42.8)**	288 (38.4)		0.260
	Unknown	5 (5.3)	56 (8.5)	61 (8.1)		
Anxiety, Confirmed Diagnosis	Yes	**60 (63.8)**	188 (28.7)	248 (33.1)	45.953 (1), < 0.001	0.248
	No/Unknown	34 (36.2)	**468 (71.3)**	502 (66.9)		
Depression, Reported	Yes	**37 (39.4)**	64 (9.8)	101 (13.5)	69.421 (2), < 0.001	
	No	49 (52.1)	**567 (86.4)**	616 (82.1)		0.304
	Unknown	**8 (8.5)**	25 (3.8)	33 (4.4)		
Depression, Confirmed Diagnosis	Yes	**42 (44.7)**	57 (8.7)	99 (13.2)	92.958 (1), < 0.001	0.352
	No/Unknown	52 (55.3)	**599 (91.3)**	651 (86.8)		
Mania/Bipolar, Reported	Yes	**37 (39.4)**	118 (18)	155 (20.7)	51.113 (2), < 0.001	0.261
	No	46 (48.9)	**524 (79.9)**	570 (76)		
	Unknown	**11 (11.7)**	14 (2.1)	25 (3.3)		
Mania/Bipolar, Confirmed Diagnosis	Yes	**21 (22.3)**	31 (4.7)	52 (6.9)	39.536 (1), < 0.001	0.230
	No/Unknown	73 (77.7)	**625 (95.3)**	698 (93.1)		
Psychosis, Reported	Yes	**64 (68.1)**	89 (13.6)	153 (20.4)	154.546 (2), < 0.001	0.454
	No	22 (23.4)	**514 (78.4)**	536 (71.5)		
	Unknown	8 (8.5)	53 (8.1)	61 (8.1)		
Psychosis, Confirmed Diagnosis	Yes	**25 (26.6)**	37 (5.6)	62 (8.3)	47.611 (1), < 0.001	0.252
	No/Unknown	69 (73.4)	**619 (94.4)**	688 (91.7)		
First Degree Family Member with Psychiatric Diagnosis	Yes	**29 (30.9)**	120 (18.3)	149 (19.9)	8.172 (2), 0.017	0.104
	No	6 (6.4)	46 (7)	52 (6.9)		
	Unknown	59 (62.8)	**490 (74.7)**	549 (73.2)		

No Reported SI includes either “No” or “Don’t Know” responses; it does not include subjects who did not respond. Bold cells are those significant by post hoc test. NS: p-value greater than 0.05. Self-injurious behavior indicates presence or absence of common behaviors associated with PWS, such as skin picking, nail pulling, rectal digging, etc. Unknown includes both “Don’t know” and no answer. Diagnosis questions only asked if symptom question answered yes. Cramer’s V: Measure of Association (Effect size ranging 0 to 1)

Along with higher rates of psychiatric comorbidities, subjects with reported SI had a higher rate and number of psychiatric hospitalization events. They also had significantly greater rates of treatment with behavioral therapies, psychotherapy, and all medication categories except stimulants and atomoxetine (Table 4).

**Table 4:** Hospitalization, Therapies, and Medication Treatment in relation to Reported SI History

		Reported SI	No Reported SI	Total Count	Pearson’s X2 (df), *p*-value	Cramer’s V
		Count (%)	Count (%)	Count (%)		
Psychiatric Hospitalization	Yes	**42 (44.7)**	59 (9)	101 (13.5)		0.353
	No	50 (53.2)	**593 (90.4)**	643 (85.7)	93.398 (2), < 0.001	
	Don’t know	2 (2.1)	4 (0.6)	6 (0.8)		
Number of Psychiatric Hospitalizations	1	**16 (17)**	28 (4.3)	44 (5.9)		0.359
	2–4	**21 (22.3)**	25 (3.8)	46 (6.1)	96.562 (3), < 0.001	
	5 or more	**4 (4.3)**	2 (0.3)	6 (0.8)		
	Unknown	53 (56.4)	**601 (91.6)**	654 (87.2)		
Applied Behavioral Analysis or Behavioral Therapy	Yes	**35 (37.2)**	153 (23.3)	188 (25.1)		
	No	28 (29.8)	**327 (49.8)**	355 (47.3)	14.464 (2), 0.001	0.139
	Unknown	31 (33)	176 (26.8)	207 (27.6)		
Hippotherapy	Yes	31 (33)	191 (29.1)	222 (29.6)	1.622 (2), NS	
	No	36 (38.3)	297 (45.3)	333 (44.4)		0.047
	Unknown	27 (28.7)	168 (25.6)	195 (26)		
Psychotherapy	Yes	**22 (23.4)**	54 (8.2)	76 (10.1)	28.665 (2), < 0.001	
	No	39 (41.5)	**430 (65.5)**	469 (62.5)		0.195
	Unknown	**33 (35.1)**	172 (26.2)	205 (27.3)		
Any Antidepressant	Yes	**31 (33)**	94 (14.3)	125 (16.7)	25.626 (2), < 0.001	
	No	39 (41.5)	**428 (65.2)**	467 (62.3)		0.185
	Unknown	24 (25.5)	134 (20.4)	158 (21.1)		
Any Antipsychotic	Yes	**39 (41.5)**	102 (15.5)	141 (18.8)	39.286 (2), < 0.001	
	No	35 (37.2)	**421 (64.2)**	456 (60.8)		0.229
	Unknown	20 (21.3)	133 (20.3)	153 (20.4)		
Any Stimulant or Atomoxetine	Yes	20 (21.3)	110 (16.8)	130 (17.3)	5.644 (2), 0.059	
	No	47 (50)	411 (62.7)	458 (61.1)		0.087
	Unknown	27 (28.7)	135 (20.6)	162 (21.6)		
Any Other Psychiatric Medication	Yes	**45 (47.9)**	146 (22.3)	191 (25.5)	30.992 (2), < 0.001	
	No	32 (34)	**394 (60.1)**	426 (56.8)		0.203
	Unknown	17 (18.1)	116 (17.7)	133 (17.7)		

No Reported SI includes either “No” or “Don’t Know” responses; it does not include subjects who did not respond. Bold cells are those significant by post hoc test. Unknown includes both “don’t know” answers and blank/unavailable data. Cramer’s V: Measure of Association (Effect size ranging 0 to 1)

## Discussion

This study provides a first look at rates of suicidal thoughts and attempts in individuals with PWS, a population whose common characteristics and comorbidities may increase vulnerability to suicidality. According to a cross-national survey of 84,850 adults across 17 countries, life-time prevalence rates of SI and SA in the general population were 9.2 and 2.7%, respectively [24]. In youth and young adults, the rates of SI and SA reportedly varied from 10.3% (SI) and 3.0% (SA) in a Canadian study [25] to 18.8% (SI) and 8.9% (SA) in the US [4]. Psychiatric comorbidities and social problems are associated with greater risk of suicide across ages groups and are all commonly present in PWS [26].

In this natural history investigation of 750 individuals with PWS, the reported lifetime prevalence of SI was 12.5% and SA was 3.3%. We identified several factors associated with a history of SI and SA in persons with PWS, including but not limited to older age, male gender, aggressive behaviors, and mental health problems. Those with reported SI or SA were predominantly adults. Notably in this study, adults with PWS have a higher prevalence of SI (22.8%) compared to rates in the general adult population, whereas youths with PWS appear to have lower rates of SI (7.7%) compared to the general population of children and adolescents. Along with having a longer period of time to experience and report suicidality, adults may also experience higher rates of SI due to reduced support after aging out of the school system. The functional status “not in school or employed” was significantly associated with reported SI in this registry (Table 2). This result highlights importance of suicidality screening and interventions especially for adults with PWS.

In this analysis comparing groups with and without a history of suicidal thoughts, individuals with reported SI were more likely to be male. This differs from general population studies in which female gender has been associated with greater SI, and male gender with higher rates of death by suicide [3]. In students, female gender was associated with higher prevalence estimates of both SI (24.1%) and SA (11%). Replication is needed to confirm the results from this study.

We had hypothesized that genetic subtype would be associated with greater rates of suicidality, but there was no significant relationship found in this study. We also hypothesized that more robust emotional/social connection (as reflected by the presence of a best friend) and financial resources (as evidenced by gross annual income) would be associated with lower rates of SI. This was not shown in our analysis, indicating that these factors should not dissuade from screening for SI.

In line with the previous reports [12, 27–29], high rates of behavioral and psychiatric comorbidity were reported in this sample (Table 3). As in general population research, in this collection of individuals with PWS, those with a reported history of SI were more likely to have depression, anxiety, bipolar disorder, or psychosis and more likely to be receiving therapeutic or pharmacologic mental health treatments. This study did not evaluate a prevalence or association of SI with trauma-related disorders, personality disorders, or eating disorders, all of which have shown association with suicide in other populations. A history of aggressive behaviors was also significantly associated with a history of suicidal thoughts, suggesting that this could be an important risk factor. Common self-injurious behaviors in PWS, such as skin-picking, don’t necessarily reflect intent to cause self-harm; this may explain how their presence in this population was not significantly associated with reported SI. Notably, the absence of any SIB appears reassuring. Investigating psychiatric family history may also be beneficial when assessing suicide risk in a person with PWS, as this was significantly more common in the group with Reported SI.

This study has several limitations. It evaluated data from a community-based sample rather than information collected at points of medical care. Data was also primarily per caregiver report rather than direct subject screening, which may limit comparison to estimates of SI and SA prevalence in other studies. As described in the Methods section above, data was also collected by the registry on a rolling basis and at different points in time. Given the significant variability among individuals with intellectual developmental disability (IDD), it is also difficult to compare our findings to other IDD populations, especially as our data did not include information about subject intellectual functioning and this can vary greatly among persons with PWS. In addition, the social and demographic makeup of our study sample should be considered when generalizing about PWS as a whole. Finally, while this study is useful as a first reflection of SI in PWS, we did not have complete data about level of intent, planning, severity, or other details of suicidality or SIB.

Suicide may be prevented with effective screening and appropriate support. Given the considerable rates of suicidal thoughts and attempts in PWS, professionals working with PWS should strongly consider screening for SI with these individuals and their caregivers. Identifying an appropriate screening method for suicidality in different levels of intellectual disability may be a challenge, however the valuable information gathered here from one broad question in the Global PWS Registry suggests that even a general suicide risk screening tool such as Ask Suicide screening Questions (ASQ, https://www.nimh.nih.gov/research/research-conducted-at-nimh/asq-toolkit-materials/) could still be meaningful. Additionally, given the close involvement of caregivers in the lives of many people with PWS, caregiver psychoeducation and screening could be considered as a potential intervention.

When considering next steps for addressing suicidality in individuals with PWS, it would be helpful to take a closer look at SI and SA with specific levels of intellectual disability and with a broader range of psychiatric comorbidities. Direct subject assessment of randomly selected individuals from the Global PWS Registry would also be a step toward better understanding the risks for and life experiences of those with PWS.

## Conclusion

This is the first study that examined the rates of reported SI and SA among individuals with PWS using a Registry Survey Data. The rates of SI and SA in adults with PWS appear higher than those reported from general population. In addition to older age, this study identified potential factors associated with suicidality, including but not limited to male sex, living, education/employment status, psychiatric symptoms and disorders, and family history of mental health diagnosis. Findings from this study need to be replicated from an independent sample and/or larger sample.

## Data Availability

The Global Prader-Willi Syndrome Registry (https://pwsregistry.org/) data that support the findings of this study are available from the Foundation for Prader-Willi Research (FPWR) (https://www.fpwr.org/the-global-pws-registry-empowering-families-advancing-research) but restrictions may apply to the availability of these data, and are not publicly available. Data are however available upon reasonable request and with permission of the Global PWS Registry Advisory Board.

## Abbreviations

PWS: Prader–Willi syndrome
SI: Suicidal ideation
SA: Suicidal attempts
UPD: Maternal uniparental disomy
SIB: Self-injurious behavior
IDD: Intellectual developmental disorders
IRB: Institutional review board

## References

[1] CDC (2020). Web-based Injury Statistics Query and Reporting System (WISQARS).

[2] Plemmons G, Hall M, Doupnik S, Gay J, Brown C, Browning W, Casey R, Freundlich K, Johnson DP, Lind C, Rehm K, Thomas S, Williams D (2018). Hospitalization for suicide ideation or attempt: 2008-2015. Pediatrics..

[3] Hedegaard H, Curtin S, Warner M: Suicide Rates in the United States Continue to Increase. in NCHS Data Brief, no 309. Hyattsville, MD 2018. 30312151

[4] Ivey-Stephenson A, Demissie Z, Crosby A, Stone D, Gaylor E, Wilkins N, Lowry R, Brown M: Suicidal Ideation and Behaviors Among High School Students — Youth Risk Behavior Survey, United States, 2019. in MMWR Supplement 2020. pp. 47–55. 10.15585/mmwr.su6901a6 PMC7440198 32817610

[5] Bachmann S. Epidemiology of suicide and the psychiatric perspective. Int J Environ Res Public Health. 2018;15(7). 10.3390/ijerph15071425. 10.3390/ijerph15071425 PMC6068947 29986446

[6] Cassidy SB, Driscoll DJ (2009). Prader-Willi syndrome. Eur J Hum Genet.

[7] Goldstone AP, Holland AJ, Hauffa BP, Hokken-Koelega AC (2008). Tauber M, speakers contributors at the second expert meeting of the Comprehensive Care of Patients with PWS. Recommendations for the diagnosis and management of Prader-Willi syndrome. J Clin Endocrinol Metab.

[8] Nicholls RD, Knepper JL (2001). Genome organization, function, and imprinting in Prader-Willi and Angelman syndromes. Annu Rev Genomics Hum Genet.

[9] Dykens EM, Cassidy SB, King BH (1999). Maladaptive behavior differences in Prader-Willi syndrome due to paternal deletion versus maternal uniparental disomy. Am J Ment Retard.

[10] Cassidy SB (1997). Prader-Willi syndrome. J Med Genet.

[11] Curfs LM, Wiegers AM, Sommers JR, Borghgraef M, Fryns JP (1991). Strengths and weaknesses in the cognitive profile of youngsters with Prader-Willi syndrome. Clin Genet.

[12] Dykens E, Shah B (2003). Psychiatric disorders in Prader-Willi syndrome: epidemiology and management. CNS Drugs.

[13] Dykens EM, Cassidy SB (1995). Correlates of maladaptive behavior in children and adults with Prader-Willi syndrome. Am J Med Genet.

[14] Bennett JA, Germani T, Haqq AM, Zwaigenbaum L (2015). Autism spectrum disorder in Prader-Willi syndrome: a systematic review. Am J Med Genet A.

[15] Descheemaeker MJ, Govers V, Vermeulen P, Fryns JP (2006). Pervasive developmental disorders in Prader-Willi syndrome: the Leuven experience in 59 subjects and controls. Am J Med Genet A.

[16] Yang L, Zhan GD, Ding JJ, Wang HJ, Ma D, Huang GY, Zhou WH (2013). Psychiatric illness and intellectual disability in the Prader-Willi syndrome with different molecular defects--a meta analysis. PLoS One.

[17] Merrick J, Merrick E, Lunsky Y, Kandel I (2005). Suicide behavior in persons with intellectual disability. ScientificWorldJournal..

[18] Walters AS, Barrett RP, Knapp LG, Borden MC (1995). Suicidal behavior in children and adolescents with mental retardation. Res Dev Disabil.

[19] Hardan A, Sahl R (1999). Suicidal behavior in children and adolescents with developmental disorders. Res Dev Disabil.

[20] Lunsky Y (2004). Suicidality in a clinical and community sample of adults with mental retardation. Res Dev Disabil.

[21] Ludi E, Ballard ED, Greenbaum R, Pao M, Bridge J, Reynolds W, Horowitz L (2012). Suicide risk in youth with intellectual disabilities: the challenges of screening. J Dev Behav Pediatr.

[22] Bohonowych J, Miller J, McCandless SE, Strong TV. The global prader-willi syndrome registry: development, launch, and early demographics. Genes (Basel). 2019;10. 10.3390/genes10090713 PMC6770999 31540108

[23] Bohonowych JE, Vrana-Diaz CJ, Miller JL, McCandless SE, Strong TV (2021). Incidence of strabismus, strabismus surgeries, and other vision conditions in Prader-Willi syndrome: data from the global Prader-Willi syndrome registry. BMC Ophthalmol.

[24] Nock MK, Borges G, Bromet EJ, Alonso J, Angermeyer M, Beautrais A, Bruffaerts R, Chiu WT, de Girolamo G, Gluzman S, de Graaf R, Gureje O, Haro JM, Huang Y, Karam E, Kessler RC, Lepine JP, Levinson D, Medina-Mora ME, Ono Y, Posada-Villa J, Williams D (2008). Cross-national prevalence and risk factors for suicidal ideation, plans and attempts. Br J Psychiatry.

[25] Sampasa-Kanyinga H, Dupuis LC, Ray R. Prevalence and correlates of suicidal ideation and attempts among children and adolescents. Int J Adolesc Med Health. 2017;29(2). 10.1515/ijamh-2015-0053. 10.1515/ijamh-2015-0053 26556839

[26] Skokauskas N, Sweeny E, Meehan J, Gallagher L (2012). Mental health problems in children with prader-willi syndrome. J Can Acad Child Adolesc Psychiatry.

[27] Soni S, Whittington J, Holland AJ, Webb T, Maina EN, Boer H, Clarke D (2008). The phenomenology and diagnosis of psychiatric illness in people with Prader-Willi syndrome. Psychol Med.

[28] Soni S, Whittington J, Holland AJ, Webb T, Maina E, Boer H, Clarke D (2007). The course and outcome of psychiatric illness in people with Prader-Willi syndrome: implications for management and treatment. J Intellect Disabil Res.

[29] Feighan SM, Hughes M, Maunder K, Roche E, Gallagher L (2020). A profile of mental health and behaviour in Prader-Willi syndrome. J Intellect Disabil Res.

